# The Interaction Network of NSm and Its Role as a Movement Protein in the Tomato Zonate Spot Virus

**DOI:** 10.3390/v17121570

**Published:** 2025-11-30

**Authors:** Xingyue Zhao, Jianbin Chen, Limin Zheng, Jiajia Tu, Xin Wang, Xiaobin Shi, Yu Zhang, Shue Sun, Jie Zhang, Xue Zheng, Deyong Zhang

**Affiliations:** 1 Hunan Plant Protection Institute, Hunan Academy of Agricultural Sciences, Changsha 410125, China; zhaoxingyue1125@163.com (X.Z.); chenjianbin89@126.com (J.C.); 2 YueLuShan Laboratory, Changsha 410125, China; 3 Microscopy Core Facility, The Biomedical Research Core Facility, Westlake University, Hangzhou 310024, China; 4 General Equipment & Autoclave Service Core Facility, The Biomedical Research Core Facility, Westlake University, Hangzhou 310024, China; 5 Yunnan Province Key Laboratory of Agricultural Biotechnology, Biotechnology and Germplasm Resource Institute, Yunnan Academy of Agricultural Sciences, Kunming 650223, China

**Keywords:** tomato zonate spot virus, self-interaction, protein–protein interactions, subcellular localization, co-localization, movement protein

## Abstract

The tomato zonate spot virus (TZSV) poses a significant threat to agriculture. Therefore, the elucidation of the functional roles and interactions of its encoded proteins is crucial for the development of effective control strategies. The aim of this study was to investigate the interaction network between the TZSV nucleocapsid (N), the non-structural M-segment (NSm) and the non-structural S-segment (NSs) proteins, with a focus on the functional characterization of the NSm protein. Yeast two-hybrid (Y2H) analysis indicated that both the N protein (N-N) and the NSm protein (NSm-NSm) exhibit self-interaction in vitro, with successful expression of all fusion proteins confirmed by Western blotting. Subsequently, we used bimolecular fluorescence complementation (BiFC) and luciferase complementation imaging (LCI) assays in epidermal cells of *Nicotiana benthamiana* to confirm that N and NSm proteins self-interact. In addition, heterologous interactions between NSs-N, N-NSm and NSs-NSm were also detected. BiFC and co-localization experiments with fusion proteins elucidated the interaction place of the cell: N-N and NSm-N interactions occurred in both the cytoplasm and nucleus, with NSm-NSm interaction occurring in the nucleus, whereas NSs-N and NSs-NSm interactions only occurred in the cytoplasm. Subcellular localization studies showed that the N protein is distributed in both the cytoplasm and the nucleus, whereas the NSm and NSs proteins are predominantly localized in the cytoplasm. In particular, NSm was found to specifically target plasmodesmata (PD) and co-localize with the known PD marker protein PDLP8. Interestingly, TZSV NSm was demonstrated to mediate the cell-to-cell movement of a cucumber mosaic virus mutant (ΔCMV-GFP) lacking its native movement protein (3a). This was evidenced by the spread of approximately 50 fluorescent foci to neighboring cells observed at 6 dpi. This study comprehensively describes the intricate interaction network between the N, NSm and NSs proteins of TZSV and clarifies their subcellular localizations within plant cells. Crucially, we provide conclusive evidence that the NSm protein of TZSV is a functional movement protein essential for facilitating viral intercellular transport which promotes viral spread within the host during systemic infection. These findings offer important insights into the infection mechanism of TZSV and provide potential targets for the control of TZSV.

## 1. Introduction

Tomato zonate spot virus (TZSV) is a dominant species of the genus *Orthotospovirus* (family *Tospoviridae*, order *Bunyavirales*) discovered in Yunnan Province, China. It is considered one of the most destructive pathogens of ornamental and vegetable crops worldwide [1,2]. To date, many species of *Orthotospovirus* have been reported in China. Meanwhile, they have also been reported in Australia, the United States, Argentina, Brazil, India, Japan, France and many other countries [3,4].

TZSV is usually transmitted by *Frankliniella palmi*, *Frankliniella schultzei* and *Frankliniella occidentalis* in a persistent and circulatory-propagative manner [5,6,7]. The virus infects important economic crops such as *Nicotiana tabacum*, *Lycopersicon esculentum*, *Capsicum annuum* and flowers, which can cause billions of dollars in economic losses every year. Since 2007, the disease caused by TZSV has spread widely in many areas in southwest China, seriously threatening local farmers’ production of tomatoes, tobacco, peppers and other cash crops [8,9].

The TZSV genome consists of three RNA strands: ssRNA-L, ssRNA-M and ssRNA-S [10,11]. ssRNA-L is the reverse negative-sense strand, encoding RNA-dependent RNA polymerase (RdRp) [12]. ssRNA-M encodes the non-structural M segment (NSm), while the complementary strand encodes the glycoproteins Gn and Gc on the phospholipid bilayer [13]. On the other hand, ssRNA-S encodes the non-structural S segment (NSs) and N segment (N). Current knowledge of the functions of TZSV proteins comes primarily from homologous studies in other *orthotospoviruses* [4,14]. TZSV-encoded NSs have been shown to be viral suppressors of RNA silencing and viral pathogenicity factors [15]. So far, the TZSV proteins have not yet been reported.

Molecular interactions between viral proteins are crucial regulatory nodes in the viral life cycle, controlling replication complex formation, virion assembly, intracellular transport and vector-mediated transmission. Several protein–protein interactions have been identified in other *orthotospoviruses*. For example, self-interaction and polymerization of TSWV N proteins were shown to be detected by yeast two-hybrid assays, and two interacting domains were identified and characterized [16]. It has been reported that in the interactions between N and NSm, the NSm protein can interact with the N protein and bind to single-stranded RNA (ssRNA) in a sequence-nonspecific manner, and bind to plasmodesmata in tomato spotted wilt virus (TSWV) [17]. In addition, fluorescence microscopy analysis of kidney cells of small hamsters revealed the interaction dynamics of the TSWV N protein [18]. Previous studies have demonstrated that the N protein interacts with various other viral proteins, such as the NSm, NSs and the glycoproteins (Gn/Gc), as confirmed in viruses like impatiens necrotic spot virus (INSV), capsicum chlorosis virus (CaCV), iris yellow spot virus (IYSV), bean necrotic mosaic virus (BNMV), chrysanthemum stem necrosis virus (CSNV) and tomato chlorotic spot virus (TCSV) [19,20,21,22,23]. Furthermore, protein self-interactions and complex interaction networks, such as those for TSWV, have also been reported [24]. However, for TZSV, the interaction network among its N, NSm and NSs proteins remains to be elucidated.

In this study, we used a combined yeast two-hybrid (Y2H) assay, BiFC, luciferase complementation imaging (LCI) assay and co-localization analysis in yeast and *N. benthamiana* to analyze self-interaction of TZSV-encoded NSs, NSm and N proteins. We found both self- and cross-interactions among the three viral proteins in *N. benthamiana*, although NSs did not show positive self-interaction by Y2H. Moreover, we identified NSm as a movement protein and also established the localizations of the viral proteins. These results provide a foundation for future study of the pathogenic mechanisms of TZSV and viral particle assembly.

## 2. Materials and Methods

### 2.1. Virus and Plant Material

A tomato plant infected with TZSV was collected from Yuanmou, Yunnan Province, and the virus was isolated, purified and inoculated into *Nicotiana benthamiana* by friction [4]. The infected leaves were stored at −80 °C as the virus source. *N. benthamiana* plants were grown in a growth chamber at 60% relative humidity, with a 16 h photoperiod at 25 °C during the day and an 8 h dark period at 20 °C during the night.

### 2.2. Preparation of Plasmids

Total RNA was extracted from the leaves of TZSV-infected *N. benthamiana* using TRIzol reagent (Invitrogen, Thermo Fisher Scientific, Carlsbad, CA, USA), and the first-strand cDNA was synthesized using the Hiscript II first strand cDNA synthesis kit (Vazyme, Nanjing, China). The primer pairs for amplifying the ORFs of the three genes encoding N (GenBank: MG656995.1), NSm (GenBank: EF552434.1) and NSs (GenBank: KC133530.1) of TZSV, respectively, were designed in the viral sequence database of GenBank. The primers used to amplify the TZSV genes are listed in Table S1. The N, NSm and NSs proteins were cloned and expressed in their full lengths.

### 2.3. Yeast Two-Hybrid (Y2H) Assays

A yeast two-hybrid system was used to confirm the interaction among the three proteins N, NSm and NSs of TZSV, according to a previous protocol [15]. The full-lengths of the genes encoding the N, NSm and NSs proteins were amplified by PCR and cloned into pGADT7/pGBKT7 vectors, respectively, for the assay. Combinations of bait and prey plasmids were co-transformed into the Y2HGold reporter strain.

### 2.4. Western Blot Analysis

In order to detect the expressions of N, NSm and NSs in yeast, total yeast protein was extracted using a yeast protein extraction reagent kit (Invent Biotechnologies, Eden Prairie, MN, USA). Monoclonal yeasts containing BD-N, BD-NSm, BD-NSs, BD, AD-N, AD-NSm, AD-NSs and AD were cultured in SD/Trp or SD/Leu liquid medium with shaking at 180 rpm, 30 °C. The total protein extracts were analyzed by SDS-PAGE and Western blot as described in a previous study [15]. Anti-Myc and anti-HA antibodies were used for the Western blot assay, and protein bands were detected using the Novex ECL chemiluminescent substrate reagent kit (Invitrogen, Thermo Fisher Scientific, Carlsbad, CA, USA).

### 2.5. BiFC Assays

To construct the plasmids for use in the BiFC assay, specific primers were used to amplify the target fragments of N, NSm and NSs (Table S1). The fragments were cloned into pCV-cYFP or pCV-nYFP vectors. The YFP-fusion constructs were transformed into *Agrobacterium tumefaciens* strain GV3101 by electroporation method. *Agrobacterium* cultures were grown overnight, pelleted through centrifugation and incubated for 2 h in infiltration buffer (100 mM MES, pH 5.2, 10 mM MgCl_2_ and 200 mM acetosyringone). *Agrobacterium* cultures carrying N-cYFP, NSm-cYFP, NSs-cYFP or cYFP (OD_600_ = 1) were mixed with an equal volume of *Agrobacterium* cultures carrying N-nYFP, NSm-nYFP, NSs-nYFP or nYFP. The mixed cultures were infiltrated individually into leaves of *N. benthamiana* plants using needleless syringes via *Agrobacterium*-mediated transient expression (*Agroinfiltration*). The BiFC assays were caried out as described previously [15].

### 2.6. Luciferase Complementation (LCI) Assay

For luciferase complementation assays (LCI), the full-length fragments of the genes encoding N, NSm and NSs were amplified and cloned into cLuc or nLuc. Subsequently, the constructs were transformed into *A. tumefaciens* strain GV3101. Positive transformants were cultured, harvested and resuspended in infiltration buffer. *Agrobacterium* cultures carrying N-cLUC, NSm-cLUC, NSs-cLUC or cLUC were mixed with equal volumes of *Agrobacterium* cultures carrying N-nLUC, NSs-nLUC, NSm-nLUC or nLUC. Then, the mixed cells were transfected into the leaves of *N. benthamiana* via *Agroinfiltration*. After 24 h of infiltration, the infiltrated leaves were sprayed with a 1 mM luciferin solution. Fluorescence images were visualized using an NEWTON7.0 Bio plus Vilber, Paris, France).

### 2.7. Subcellular Localization

To investigate the subcellular localization of each of the TZSV viral proteins, full-length N, NSm and NSs were amplified and ligated into pCV-GFP or pCV-mCherry plasmids after digestion with *Kpn* I and *Bam*H I restriction enzymes. Subsequently, N-GFP, NSm-GFP, NSs-GFP, N-mCherry, NSm-mCherry and NSs-mCherry fusion constructs were generated. The primers used are listed in Table S1. These constructs were separately transformed into *A. tumefaciens* strain GV3101. The subcellular localization and co-localization assays were caried out as reported in a previous study [15].

### 2.8. Complementation Assay of the NSm Protein with a CMV Movement Protein Deletion Mutant

To further investigate the function of the NSm protein in mediating viral intercellular movement, the full-length NSm was amplified and ligated into the pCV-3HA plasmid following digestion with *Kpn* I and *Bam*H I restriction enzymes. Subsequently, a TZSV NSm-3HA (TZSV NSm) fusion construct was generated. The primers used for amplification are listed in Table S1. This construct was transformed into *A. tumefaciens* strain GV3101.

A complementation assay was conducted to determine whether the TZSV NSm protein could facilitate the intercellular movement of cucumber mosaic virus (CMV). This assay utilized an infectious clone of CMV (ΔCMV-GFP), in which the native movement protein gene (3a) had been replaced by the GFP gene. As a positive control, leaves were similarly infiltrated with *Agrobacterium* expressing TSWV NSm, a previously reported movement protein [25,26]. The complementation analysis was carried out as reported in a previous study [27].

### 2.9. Statistical Analysis

Unless otherwise stated, all experiments were performed in at least 3 biological replicates in all cases, with each repetition including at least 3 samples. Before statistical analysis, all data were first tested for normal distribution using the Shapiro–Wilk test. GFP fluorescence signals were statistically analyzed by *t*-tests using GraphPad Prism 8.0 software. Values with * (*p* < 0.05) and ** (*p* < 0.01) were considered statistically significant.

## 3. Results

### 3.1. N, NSm and NSs Interact with Each Other In Vitro

**Figure 1:**
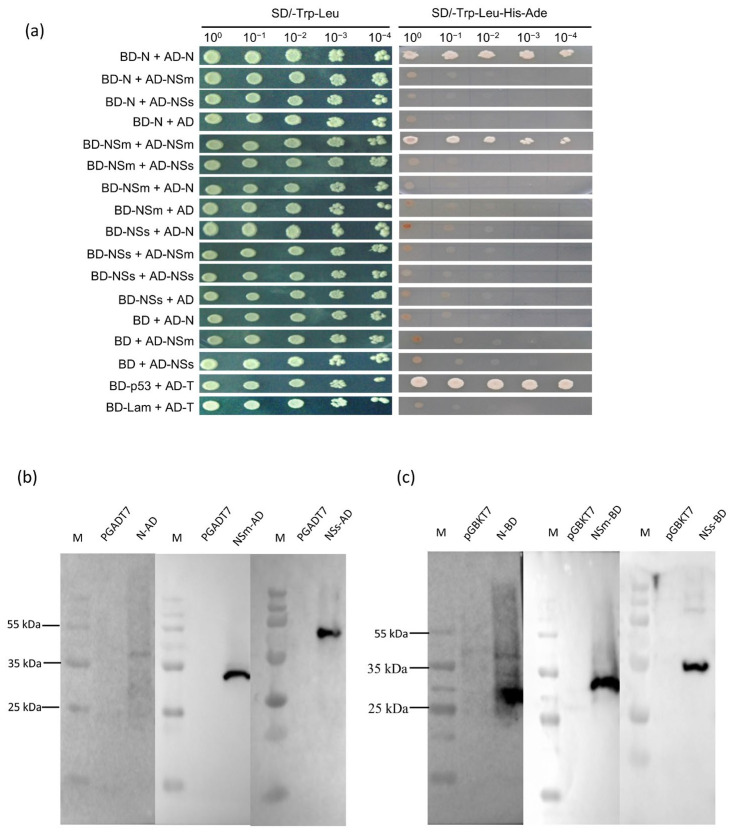
N, NSm and NSs interacts with each other in yeast two-hybrid analysis. (**a**) Yeast two-hybrid (Y2H) assay was performed to determine self-interaction between N and NSm. Y2HGold yeast cells co-expressing AD-N, AD-NSm, AD-NSs or AD empty vector (pGADT7) were used to assay for the interaction with BD-N, BD-NSm, BD-NSs or BD empty vector (pGBKT7). AD-T+BD-p53 or AD-T+BD-Lam were used as the positive and the negative controls, respectively. Y2HGold cells were diluted 10^0^ to 10^−4^ and plated onto QDO (SD−Trp−Leu−His−Ade) medium. (**b**,**c**) Western blot analysis confirmed the correct expression of yeast expression vectors in yeast cells. The recombinant prey expression vector and recombinant bait expression vector were analyzed using HA-tag and c-Myc-tag antibodies, respectively. Empty vectors were used as controls.

Yeast two-hybrid (Y2H) assays were performed to identify interactions between the proteins N, NSm and NSs, including possible self-interactions. As shown in Figure 1a, yeast cells co-expressing BD-N/AD-N or BD-NSm/AD-NSm grew on the high stringent selective medium SD/-Leu/-Trp/-His/-Ade, similar to the positive control (BD-p53/AD-T). These results provide preliminary evidence that both the TZSV N protein and the NSm protein self-interact in vivo within the yeast system.

The expression of the fusion proteins in Y2HGold yeast was verified by Western blot to support the Y2H interaction data. Total protein extracts were analyzed by SDS-PAGE followed by immunoblotting with anti-c-Myc (for BD fusions) and anti-HA (for AD fusions) antibodies. As shown in Figure 1b,c, the bands corresponding to the predicted sizes were detected for BD-N, BD-NSm, BD-NSs, AD-N, AD-NSm and AD-NSs. This confirms the successful expression of all six fusion proteins and is consistent with the observed Y2H interaction results.

### 3.2. N, NSm and NSs Interact with Each Other in N. benthamiana

**Figure 2:**
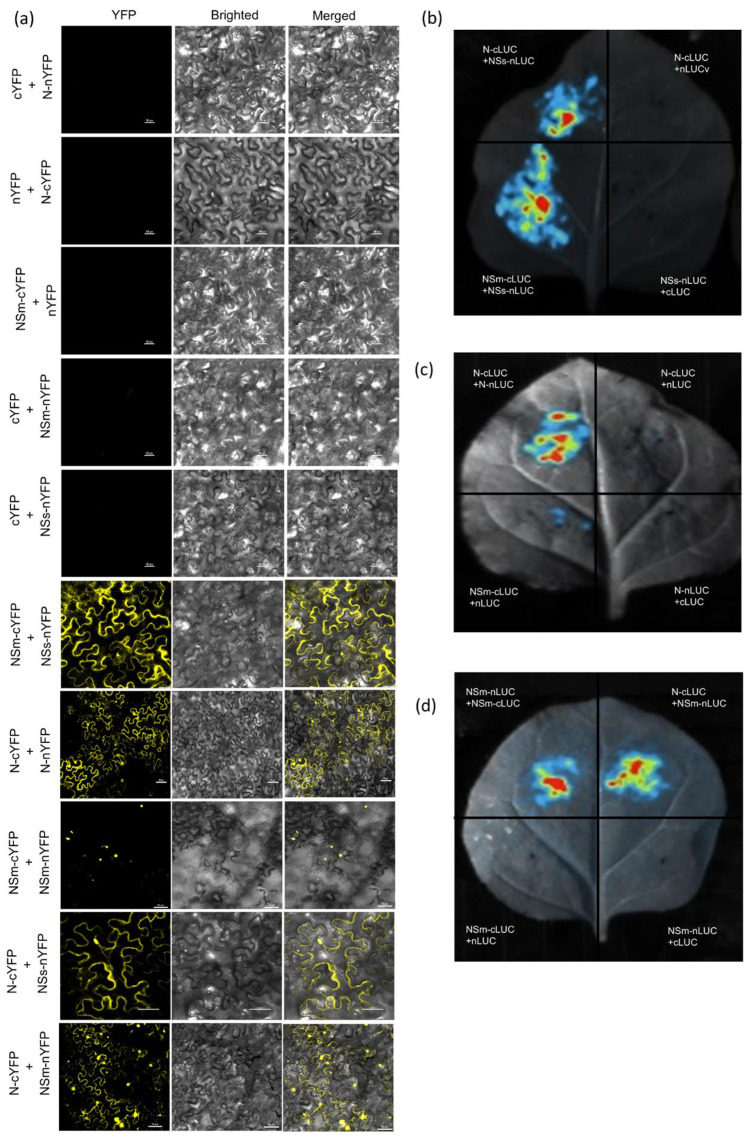
The interaction between N, NSm and NSs were confirmed in *N. benthamiana* byBiFC and LCI assays. (**a**) BiFC assay of the interaction among N, NSm and NSs in *N. benthamiana*. N-cYFP was co-expressed with N-nYFP, NSs-nYFP, NSm-nYFP or nYFP. NSm-cYFP was co-expressed with NSm-nYFP, NSs-nYFP or nYFP. N-nYFP was co-expressed with cYFP. NSm-nYFP was co-expressed with cYFP. NSs-nYFP was co-expressed with cYFP. Recombinant expression vectors combined with unfused empty vectors (pCV-cYFP and pCV-nYFP) were used as negative controls. YFP signals in *N. benthamiana* leaves were recorded at 3 dpi. Bars: 20 μm. (**b**–**d**) Interaction of N with itself, NSm with itself, N with NSm, NSm with NSs and NSs with N analyzed by LCI assay in *N. benthamiana* leaves. N-cLUC, NSm-cLUC or cLUC were co-expressed with N-nLUC, NSs-nLUC, NSm-nLUC or nLUC, respectively. Recombinant expression vectors combined with unfused empty vectors (cLUC and nLUC) were used as negative controls. Luciferase activity was detected at 24 hpi.

We further investigated the interactions between the N, NSm and NSs proteins in planta using BiFC and LCI. The N, NSm and NSs proteins tagged with YFP (nYFP/cYFP) or split luciferase (nLuc/cLuc) fragments were generated and transiently co-expressed in *N. benthamiana* leaves via *Agroinfiltration*. For BiFC, fluorescence microscopy showed positive interaction between N-N and NSm-N in both the cytoplasm and nucleus, with the NSm-NSm interaction localized to the nucleus, whereas NSs-NSm and NSs-N interactions were detected in the cytoplasm (Figure 2a). Consistent with these observations, LCI assays showed significantly higher luciferase activity for N-N, NSm-NSm, NSs-NSm, NSm-N and NSs-N interaction pairs compared to the negative controls (Figure 2b–d), confirming these interactions within plant cells.

### 3.3. Subcellular Localization and Co-Localization Analysis of TZSV N, NSm and NSs Proteins in N. benthamiana

To investigate the subcellular distribution of the TZSV N, NSm and NSs proteins and to visualize their potential interactions in planta, we transiently expressed the fusion proteins in *N. benthamiana* epidermal cells for localization and co-localization analyses.

**Figure 3:**
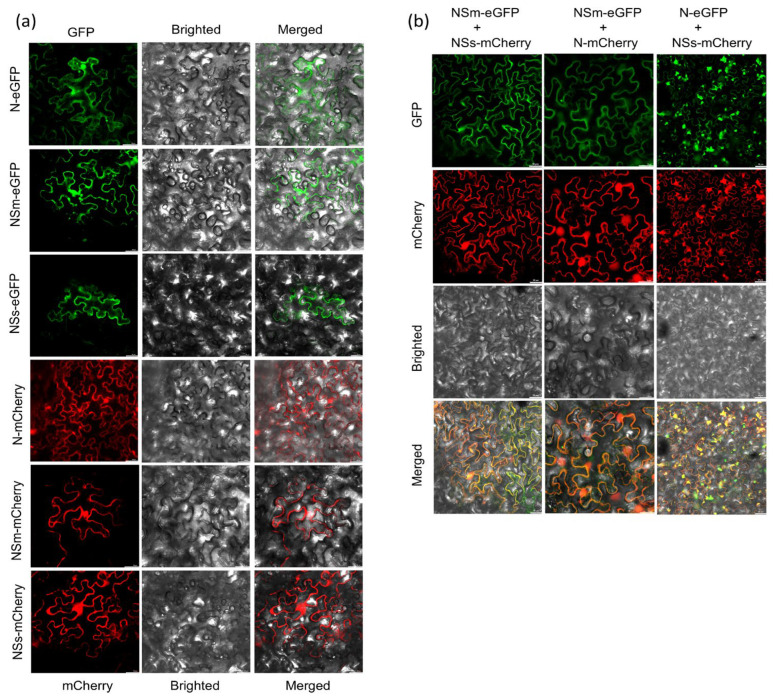
The subcellular localization and co-localization analysis of N, NSm and NSs in *N. benthamiana*. (**a**) Subcellular localization of N, NSm and NSs proteins within *N. benthamiana* leaf epidermal cells agroinfiltrated with N-eGFP, NSm-eGFP, NSs-eGFP or N-mCherry, NSm-mCherry, NSs-mCherry by GFP or RFP assay.GFP and RFP fluorescence signals were recorded by confocal microscope at 3 dpi. Bars: 50 μm. (**b**) Confocal micrographs of *N. benthamiana* co-expressing NSm-eGFP and NSs-mCherry, N-mCherry and NSm-eGFP or N-eGFP and NSs-mCherry at 3 dpi. Bars: 50 μm. The first column shows the subcellular localization of NSm and NSs determined by the GFP and mCherry channels, respectively, and shows the subcellular localization of NSm and NSs determined by merging the GFP and mCherry channels. The second column shows the subcellular localization of NSm and N determined by the GFP and mCherry channels, respectively, and shows the subcellular localization of NSm and N determined by merging the GFP and mCherry channels. The third column shows the subcellular localization of N and NSs determined by the GFP and mCherry channels, respectively, and shows the subcellular localization of N and NSs determined by merging the GFP and mCherry channels.

Confocal microscopy revealed distinct subcellular localization patterns for each protein when expressed individually (Figure 3a). The N protein was observed to be widely distributed throughout the cytoplasm and the nucleus. Conversely, the NSm and NSs proteins were predominantly found in the cytoplasm. Furthermore, co-localization experiments were subsequently conducted to investigate the possibility of interaction among these proteins. Co-expression of NSm-eGFP and NSs-mCherry resulted in a significant overlap of their fluorescent signals within the cytoplasm (Figure 3b). Similarly, substantial co-localization was observed in the cytoplasm when N-eGFP and NSs-mCherry were co-expressed. Notably, the co-expression of NSm-eGFP and N-mCherry showed overlapping signals in both the cytoplasm and the nucleus.

### 3.4. The TZSV NSm Protein Functions as a Movement Protein

**Figure 4:**
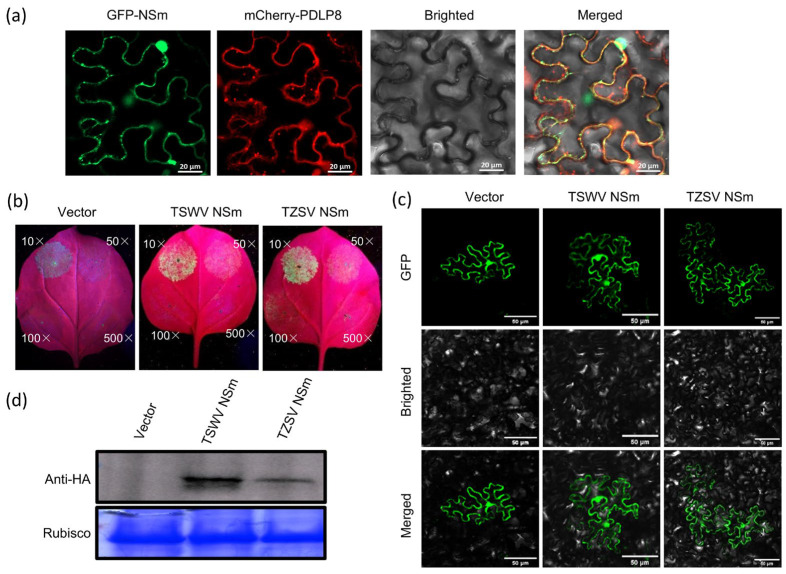
The NSm protein could complement the movement capacity of TZSV and TSWV movement proteins between epidermal cells in *N. benthamiana* leaves. (**a**) Complementary analysis of confocal micrographs of *N. benthamiana* co-expressing NSm-GFP and PDLP8-mCherry at 3 dpi. The first and second columns show the subcellular localization of NSm and PDLP8 determined by the GFP and mCherry channels, respectively. The third column shows the bright field. The fourth column shows the subcellular localization of NSm and PDLP8 determined by merging the GFP and mCherry channels. Bars: 20 μm. (**b**) *N. benthamiana* leaves were infiltrated with *Agrobacterium tumefaciens* GV3101 cultures (10-, 50-, 100- or 500-fold dilution of OD_600_ = 1.0). Complementary analysis of TZSV NSm and tomato spotted wilt virus (TSWV) NSm as a positive control under UV light. (**c**) Complementary analysis of TZSV NSm or TSWV NSm and cucumber mosaic virus movement protein deficiency mutant under a confocal microscope. GFP fluorescence signals were recorded by confocal microscope at 6 dpi. Bars: 50 μm. (**d**) Western blot detection of TZSV NSm and TSWV NSm expressed in epidermal cells of *N. benthamiana* leaves.

To investigate if the NSm protein targets PD within the cell wall, we co-expressed NSm-GFP and PDLP8-mCherry in *N. benthamiana* leaves via *Agroinfiltration*. We used PDLP8-mCherry (*Arabidopsis thaliana* plasmodesmata-located protein 8) as it is a well-established marker for PD localization [28]. The plant leaves were harvested at 48 hpi and the subcellular localization patterns of these two fusion proteins were examined using a confocal microscope. The results revealed that the NSm-GFP fusion protein accumulated in green, fluorescent punctate structures within the cytoplasm and near the cell wall (Figure 4a). The PDLP8-mCherry fusion protein produced red fluorescent signals at the cell wall and in the cytoplasm, although it did not form the large punctate structures observed in NSm-GFP. Importantly, co-localization of NSm-GFP and PDLP8-mCherry fluorescence signals were observed in the cytoplasm and at the cell wall (Figure 4a). As expected for a movement protein, NSm was found to localize to the plasmodesmata.

To further check whether the NSm protein functions as a movement protein mediating viral cell-to-cell movement via PD, we co-infiltrated *N. benthamiana* leaves with *Agrobacterium* cultures expressing NSm together with ΔCMV-GFP. As shown in Figure 4b,c, similar to the positive control TSWV NSm, the TZSV NSm protein was observed to mediated the cell-to-cell movement of the movement-deficient CMV mutant via the PD. At 6 dpi, a total of 45 of the 50 fluorescent signals were detected in the leaves co-expressing TZSV NSm and ΔCMV-GFP (Table 1). However, in the leaves co-expressing ΔCMV-GFP and either TZSV NSm or TSWV NSm, the GFP signal was observed to have spread into adjacent cells, indicating successful cell-to-cell movement. In contrast, the fluorescence signal in the control sample remained confined within the initially infiltrated cells (Figure 4c).

## 4. Discussion

In plant virus research, interactions among viral proteins impact various facets, such as viral particle structure and assembly, nucleic acid replication, regulation of gene expression, transmission mechanisms, viral movement, pathogenicity and protein localization. For instance, self-interaction of the cucumber mosaic virus (CMV) 2b protein plays a vital role in the suppression of RNA silencing and the induction of viral disease symptoms [29]; self-interaction of the rice stripe virus (RSV) NP protein is essential for its localization in the nucleus [30]; self-interaction of the potato mop-top virus (PMTV) TGB1 protein is needed for cell-to-cell movement [31]; and the self-interaction of the RSV NS3 protein proves indispensable for countering host defenses by suppressing RNA silencing [32]. In addition, it was shown the self-interaction of the watermelon silver mottle virus (WSMV) NSs protein is crucial for maintaining normal functions of RNA-silencing suppression and pathogenicity [33]. Rice grassy stunt virus (RGSV) encodes P5 and P3 proteins that interact with each other. Moreover, the P5 protein can self-interact. It has been demonstrated that the co-expression of P5 and P3 can enhance the pathogenicity of potato virus X in *N. benthamiana* [34,35].

Previous studies found self-interaction and cross-interactions of viral proteins belonging to the family *tospoviridae*. For example, the self-interaction of NSm, N, G_N_, G_C_ and NSs, as well as the interactions between the different TSWV proteins, have been demonstrated [24]. BiFC assays also proved the self-interaction of the PVWV N protein in host plants [19]. A study has proven that the N and NSm proteins of INSV not only interact with each other, but also self-interact [20]. Another study also demonstrated positive interaction between N and NSs, as well as between N and Gn proteins of CaCV [21]. In their study of IYSV proteins, Tripathi et al. also proved that the N and NSm proteins of the virus do interact with each other [22]. Interaction between viral proteins is a common phenomenon. In this study, we used multiple protein interaction assays including BiFC, Y2H, LCI and co-localization analysis to systematically investigate the interaction network among the three proteins (N, NSm and NSs) encoded by TZSV. Our data indicated that the TZSV-encoded NSm and N proteins self-interact, and this was confirmed by BiFC and LCI. However, self-interaction of the NSs protein was not detected (Supplementary Figure S1).

Additionally, we found cross-interactions among the three viral proteins through BiFC, LCI assays and co-localization in *N. benthamiana*. We analyzed the intracellular localization of these viral proteins in the host plant and found that the N and NSm proteins localized to the cell periphery and nucleus. In contrast, NSs could not be detected in the nucleus, consistent with the inability to establish its self-interaction using the nucleus-based yeast two-hybrid assay. This result is consistent with a recent study revealing the self-interaction of TSWV NSs, which localizes to the cytoplasm [24]. These findings provide a basis for further research on the NSs protein. TZSV structural (N) and non-structural (NSs, NSm) proteins accumulated in the cytoplasm when transiently co-expressed. Notably, the N and NSm proteins were observed to co-localize in both the cytoplasm and the nucleus. This finding is consistent with a previous study in which immunogold labeling shows an association of TSWV NSm protein with nucleocapsid aggregates and PD of infected plant cells [36]. Another study also demonstrated an interaction between the two proteins [17]. The interaction between the N protein and the NSm protein provides a molecular basis for understanding the assembly of viral particles and the factors that determine the viral spread [17,37,38]. Interestingly, cytoplasmic localization of viral proteins is common among *tospoviruses* [20,21,22,23,24]. So far, studies on *Tospoviruses* have showed that their proteins replicate in the cytoplasm of infected host plant cells [39,40]. Therefore, our data on the intracellular co-localization of TZSV-encoded proteins suggest a possible strategy that TZSV also replicates in the cytoplasm. Moreover, We have identified TZSV NSm as a movement protein, and it has been shown to co-localize with the plasmodesmata-associated protein PDLP8 in *N. benthamiana* [28]. It was also discovered that the TZSV NSm fusion protein is capable of translocating between epidermal cells. Moreover, NSm could complement a cucumber mosaic virus mutant that lacks its movement protein (ΔCMV-GFP), thereby enabling this mutant to move from one cell to another. NSm was shown in a previous study to function as a cell-to-cell movement protein for TSWV [25]. Therefore, it is inferred that the self-interaction of NSm as a movement protein is critical for the assembly of the viral movement complex, which in turn facilitates the intercellular spread of the virus in plants. In addition to its well-characterized role as a PD-associated movement protein, our findings reveal that NSm undergoes self-interaction within the nucleus, suggesting a multifaceted mechanism essential for both viral movement and replication. This finding aligns with observations regarding the P5 protein of pear chlorotic leaf spot-associated virus (PCLSaV), a protein known to complement the movement function of the PVXΔP25 mutant. Notably, P5 self-interactions are not only localized to the nucleus (forming prominent perinuclear aggregates), but also manifest as discontinuous parallel punctae at the PD and distribute along the ER network [41]. These striking parallels suggest that nuclear-localized self-interaction might represent a conserved strategy among specific viral movement proteins, potentially serving to coordinate the interplay between viral replication and intercellular transport. Previous research has established that the endoplasmic reticulum (ER) membrane transport system is a critical and direct pathway for the intercellular movement of the NSm protein and TSWV [42]. Therefore, the specific contribution of the plant ER membrane transport system to the movement of the NSm protein and the intercellular transport of TZSV warrants further investigation. In summary, our study identified self-interaction of TZSV-encoded NSs, NSm and N proteins, as well as cross-interactions among these three viral proteins, Meanwhile, we have revealed the function of NSm as a movement protein. In addition to advancing our molecular understanding of how TZSV causes disease and spreads within its host plants, these discoveries also lay the foundation for future antiviral interventions by identifying new potential targets and providing a solid theoretical framework.

## 5. Conclusions

In this study, we systematically elucidated the interaction network among three key proteins of TZSV N, NSm and NSs, and clarified the biological function of NSm. We demonstrated that both the N and NSm proteins exhibit self-interaction. Concurrently, we identified a series of hetero-interactions, including N-NSm, N-NSs and NSm-NSs. Subcellular localization and co-localization analysis provided spatial evidence for these interactions at the cellular level, revealing that the N-NSm interaction occurs in both the cytoplasm and the nucleus, while the N-NSs and NSm-NSs interactions are primarily localized to the cytoplasm. This provides a framework for understanding their coordinated functions during the viral infection. Furthermore, we established that the NSm protein specifically targets the PD, the critical channels for intercellular transport in plants. Most importantly, functional assays confirmed TZSV NSm as the key movement protein that mediates viral cell-to-cell transport within the host plant. Collectively, these discoveries advance our knowledge of the molecular mechanics of the TZSV infection cycle and provide a solid theoretical basis for the future development of antiviral strategies that target and inhibit viral movement.

## Figures and Tables

**Table 1:** Movement of TZSV NSm between epidermal cells in *N. benthamiana* leaves at 6 dpi.

Constructs	No. of Loci Examined	No. of Loci with ^a^ Single Cell	No. of Loci with More Than 2 Cells	*p*-Value
Vector	50	50 ^a^	0	
TSWV NSm	50	4	46	*p* < 0.05 ^b^
TZSV NSm	50	5	45	*p* < 0.05

^a^ Loci with single cells expressing GFP fluorescence. ^b^ *p*-values determined using unpaired two-tailed Student’s *t*-test.

## Data Availability

Data are contained within the article and Supplementary Materials.

## Supplementary Materials

The following supporting information can be downloaded at: https://www.mdpi.com/article/10.3390/v17121570/s1, Table S1: PCR primers used in the experiments. Figure S1 The self-interaction of NSs in *N. benthamiana*, determined by BiFC and LCI assays.
