# Supplementary material for: The Interaction Network of NSm and Its Role as a Movement Protein in the Tomato Zonate Spot Virus
Source: Viruses. 2025 Nov 30;17(12):1570. doi: 10.3390/v17121570 (PMC12737781; doi:10.3390/v17121570)
Supplement: Supplementary file 1 [file viruses-17-01570-s001.zip › SCI-Table-.pdf]

Table S1 PCR primers used in the experiment

Primers	Sequences(5′-3′)	Cleavage sites	Genes/Constructs
N-F	ATGTCTAACGTCCGGAGTTTAACA		N
N-R	TTAAAAAGACAGATCATTGCTGCTC		
NSm-F	ATGTCTCGCATTACTAACGTCCTCA		NSm
NSm-R	TTAGAAATCTAATGTGTTGTCAACATCTATC		
NSs-F	ATGTCTACTGCAAAGATGTCTGCTG		NSs
NSs-R	CTAAGCAGTTTGAACCTTTTCCTCA		
N-AD-F	5′-CGGAATTCATGTCTAACGTCCGGAGTTTAA-3′	*EcoR*I	AD-N
N-BD-R	5′-CGGGATCCGTTAAAAAGACAGATCATTGCTG-3′	*BamH*I	BD-N
NSm-AD-F	5′-CGCATATGATGTCTCGCATCACTAACGTC-3′	*Nde*I	AD-NSm
NSm-BD-R	5′-CGGAATTCTTAGAAATCCAATGTGTTGTCAAC-3′	*EcoR*I	BD-NSm
NSs-AD-F	5′-CGCATATGATGTCTACTGCAAAGATATCTGC-3′	*Nde*I	AD-NSs
NSs-BD-R	5′-CGGAATTCCTAAGCAGTTTGAACCTTTTCC-3′	*EcoR*I	BD-NSs
N-cYFP-F	GGAGTCGACGCACAGGGTACCATGTCTAACGTCCG GAGTTTAACA	*Kpn*I	pCV-N-cYFP
N-cYFP-R	TTCGAGCTCGCCTGGGGATCCTTACTTAAAAAGAC AGATCATTGCTGCTC	*Bam*HI	
N-nYFP-F	GGAGTCGACGCACAGGGTACCATGTCTAACGTCCG GAGTTTAACA	*Kpn*I	pCV-N-nYFP
N-nYFP-R	TTCGAGCTCGCCTGGGGATCCTTAAAAAGACAGAT CATTGCTGCTC	*Bam*HI	
NSm-cYFP-F	GGAGTCGACGCACAGGGTACCATGTCTCGCATTAC TAACGTCCTCA	*Kpn*I	pCV-NSm -cYFP
NSm-cYFP-R	TTCGAGCTCGCCTGGGGATCCTTACTTAGAAATCTA ATGTGTTGTCAACATCTATC	*Bam*HI	

Continue Table S1 PCR primers used in the experiment

Primers	Sequences	Cleavage sites	Genes/Constructs
NSm-nYFP-F	GGAGTCGACGCACAGGGTACCATGTCTCGCATTACTAACGTCCTCA	*Kpn*I	pCV-NSm-nYFP
NSm-nYFP-R	TTCGAGCTCGCCTGGGGATCCTTAGAAATCTAATGTGTTGTCAACATCTATC	*Bam*HI	
NSs-cYFP-F	GGAGTCGACGCACAGGGTACCATGTCTACTGCAAAGATGTCTGCTG	*Kpn*I	pCV-NSs-cYFP
NSs-cYFP-R	TTCGAGCTCGCCTGGGGATCCCTAAGCAGTTTGAACCTTTTCCTCA	*Bam*HI	
NSs-nYFP-F	GGAGTCGACGCACAGGGTACCATGTCTACTGCAAAGATGTCTGCTG	*Kpn*I	pCV-NSs-nYFP
NSs-nYFP-R	TTCGAGCTCGCCTGGGGATCCCTAAGCAGTTTGAACCTTTTCCTCA	*Bam*HI	
N-mNG-F	CATTTACGAACGATAGGGCCCATGTCTAACGTCCGGAGTTTAACA	*Apa*I	pAI-N-mNG
N-mNG-R	TCCACCAGAACCTCCGGATCCTTAAAAAGACAGATCATTGCTGCTC	*Bam*HI	
N-mCherry-F	CATTTACGAACGATAGGGCCCATGAGCAAGGGCGAGG	*Apa*I	pAI-N-mCherry
N-mCherry-R	CTCGCCCTTGCTCATGGATCCTTACTTGTAAAGCTCG	*Bam*HI	
NSm-mNG-F	CATTTACGAACGATAGGGCCCATGTCTCGCATTACTAACGTCCTCA	*Apa*I	pAI-NSm-mNG
NSm-mNG-R	TCCACCAGAACCTCCGGATCCTTAGAAATCTAATGTGTTGTCAACATCTATC	*Bam*HI	
NSm-mCherry-F	CATTTACGAACGATAGGGCCCATGTCTCGCATTACTA	*Apa*I	pAI-NSm-mCherry
NSm-mCherry-R	CTCGCCCTTGCTCATGGATCCAGAAATCTAATGTGTT	*Bam*HI	
NSs-mNG-F	CATTTACGAACGATAGGGCCCATGTCTACTGCAAAGATGTCTGCTG	*Apa*I	pAI-NSs-mNG
NSs-mNG-R	TCCACCAGAACCTCCGGATCCCTAAGCAGTTTGAACCTTTTCCTCA	*Bam*HI	
NSs-mCherry-F	CATTTACGAACGATAGGGCCCATGTCTACTGCAAAGA	*Apa*I	pAI-NSs-mCherry
NSs-mCherry-R	TCGCCCTTGCTCATGGATCCAGCAGTTTGAACCTTTTC	*Bam*HI	

Continue Table S1 PCR primers used in the experiment

Primers	Sequences	Cleavage sites	Genes/Constructs
N-nLUC-F	ACGGGGGACGAGCTCGAGCTCATGTCTAACGTCCGGAGTTTAACA	*Sac*I	P1300-N-nLUC
N- nLUC -R	GTATGGGTAGTCGACGTCGACTTAAAAAGACAGATCATTGCTGCTC	*Sal*I	
N-cLUC-F	GACGAGCTCGGTACCGGTACCATGTCTAACGTCCGGAGTTTAACA	*Kpn*I	P1300-N-cLUC
N-cLUC-R	CGAGATCTGGTCGACGTCGACTTAAAAAGACAGATCATTGCTGCTC	*Sal*I	
NSm-nLUC-F	ACGGGGGACGAGCTCGAGCTCATGTCTCGCATTACTAACGTCCTCA	*Sac*I	P1300-NSm-nLUC
NSm-nLUC-R	GTATGGGTAGTCGACGTCGACTTAGAAATCTAATGTGTTGTCAACATCTATC	*Sal*I	
NSm-cLUC-F	GACGAGCTCGGTACCGGTACCATGTCTCGCATTACTA	*Kpn*I	P1300-NSm-cLUC
NSm-cLUC-R	CGAGATCTGGTCGACGTCGACAGAAATCTAATGTGTT	*Sal*I	
NSs- nLUC -F	ACGGGGGACGAGCTCGAGCTCATGTCTACTGCAAAGATGTCTGCTG	*Sac*I	P1300-NSs-nLUC
NSs- nLUC -R	GTATGGGTAGTCGACGTCGACCTAAGCAGTTTGAACCTTTTCCTCA	*Sal*I	
NSs- cLUC -F	GACGAGCTCGGTACCGGTACCATGTCTACTGCAAAGA	*Kpn*I	P1300-NSs-cLUC
NSs- cLUC -R	CGAGATCTGGTCGACGTCGACAGCAGTTTGAACCTTTTC	*Sal*I	
NSm-3HA-F	GCCGTCGACGCACAGGGTACCATGTCTCGCATTACTAACGTCCTCA	*Kpn*I	pCV-NSm-3HA
NSm-3HA-R	GTATGGGTAGCCTGGGGATCCGAAATCTAATGTGTTGTCAACATCTATC	*Bam*HI	
