# Supplementary material for: The Interaction Network of NSm and Its Role as a Movement Protein in the Tomato Zonate Spot Virus
Source: Viruses. 2025 Nov 30;17(12):1570. doi: 10.3390/v17121570 (PMC12737781; doi:10.3390/v17121570)

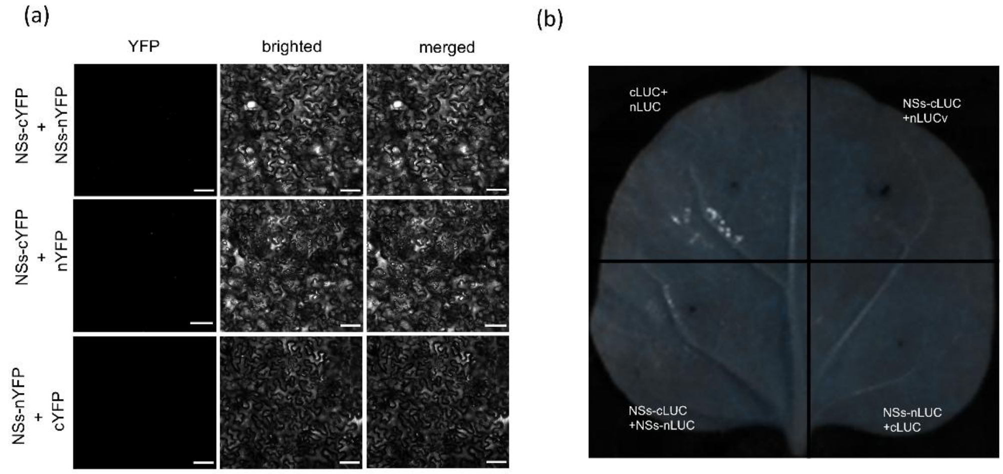

Supplementary Figure S1 The self-interaction of NSs in *N. benthamiana* by BiFC and LCI assays. (a)BiFC assays were performed in *N. benthamiana* to examine the self-interaction of NSs. NSs-cYFP was coexpressed with NSs-nYF or nYFP, NSs-nYFP was coexpressed with cYFP. Recombinant expression vectors combined with unfused empty vectors (pCV-cYFP and pCV-nYFP) were used as negative controls. YFP signals in *N. benthamiana* leaves were recorded at 3 dpi. Bars: 20 μm. (b-d) Interaction of NSs itself analyzed by luciferase complementation assay in *N. benthamiana* leaves. NSs-cLUC, or cLUC was co-expressed with NSs- nLUC or nLUC, respectively, Luciferase activity was detected at 24 hpi.

Raw images

Figure 4d. Western blotting detection of TZSV NSm and TSWV NSm expressed in epidermal cells of *N. benthamiana* leaves

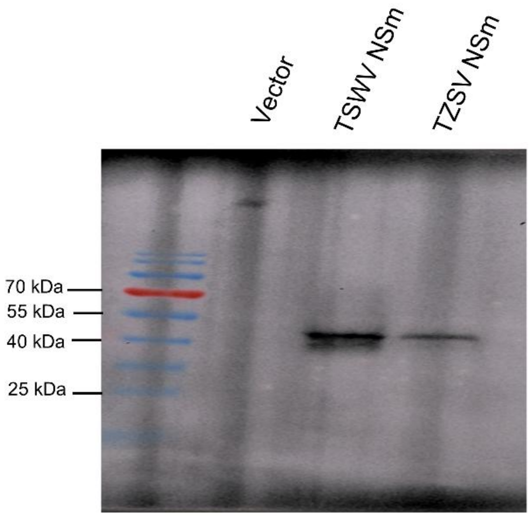

Supplement: Supplementary file 1 [file viruses-17-01570-s001.zip › viruses-3957903-supplementary.pdf]
